# IFN-*γ* Correlations with Pain Assessment, Radiological Findings, and Clinical Intercourse in Patient after Lumbar Microdiscectomy: Preliminary Study

**DOI:** 10.1155/2020/1318930

**Published:** 2020-10-13

**Authors:** Piotr Kamieniak, Joanna M. Bielewicz, Cezary Grochowski, Jakub Litak, Agnieszka Bojarska-Junak, Marzena Janczarek, Beata Daniluk, Tomasz Trojanowski

**Affiliations:** ^1^Department of Neurosurgery, Medical University of Lublin, Poland; ^2^Department of Neurology, Medical University of Lublin, Poland; ^3^Laboratory of Virtual Man, Department of Anatomy, Medical University of Lublin, Poland; ^4^Department of Clinical Immunology, Medical University of Lublin, Poland; ^5^Department of Neuroradiology and Interventional Radiology, Medical University of Lublin, Poland; ^6^Institute of Psychology, Marie Curie-Skłodowska University in Lublin, Poland

## Abstract

**Objectives:**

We investigated the influence of pain decrease after lumbar microdiscectomy on the interferon gamma (IFN-*γ*) serum level in patients with lumbar disc herniations. The study challenges the mechanism of sciatica pain and the role of IFN-*γ* in radicular pain development. *Material and Methods*. We performed clinical and immunoenzymatic assessment in a group of 27 patients with lumbar radicular pain due to disc herniations before and 3 months after surgery. Clinical status was assessed with the use of the Numeric Rating Scale (NRS), the Pain Rating Index and Pain Intensity Index of McGill Pain Questionnaire (SF-MPQ), the Oswestry Disability Index (ODI), and Beck Depression Inventory (BDI). The plasma concentrations of IFN-*γ* were ascertained by an immunoenzymatic method.

**Results:**

We observe significant correlations between the results of the pain in the back region assessment NRS back scale after the surgery with the level of IFN-*γ* before the procedure (*r*_*s*_ = 0.528; *p* = 0.008) and after the procedure (*r*_*s*_ = 0.455; *p* = 0.025). These are moderate and positive correlations—the decrease in pain is correlated with the lower IFN-*γ* level. Additionally, there are significant correlations between the results of the PRI scale and the IFN-*γ* level. The PRI score before surgery correlates positively with IFN-*γ* after surgery (*r*_*s*_ = 0.462; *p* = 0.023), and the PRI score after surgery correlates positively with IFN before surgery (*r*_*s*_ = 0.529; *p* = 0.005) and after surgery (*r*_*s*_ = 0.549; *p* = 0.003). All correlations are moderate in severity—severe pain before surgery correlates with a higher level of IFN-*γ* after surgery and also higher IFN-*γ* before surgery. There were significant differences in the IFN-*γ* level before (*Z* = −2.733; *p* = 0.006) and after (*Z* = −2.391; *p* = 0.017) surgery in the groups of patients with and without nerve compression. In the group of patients with nerve compression, the level of IFN-*γ* before and after surgery was lower.

**Conclusions:**

Less pain ratio after operation correlates with the level of IFN-*γ*. In the group of patients without significant nerve compression confirmed by MRI scans, the level of IFN-*γ* before and after surgery was higher than that in the group with nerve root compression.

## 1. Introduction

Peripheral and central sensitization leads to a reduction of the pain threshold. It is also responsible for acute and chronic radicular pain as well as nerve irritation. Evidence indicates the proinflammatory cytokines' essential role in the regulation of nociceptive phenomenons [1–3]. The key molecule controlling and modulating pain processes represents IFN-*γ*, an important mediator and modulator of neuroinflammation [4]. There are numerous reports pointing at spontaneous pain occurrence as a side effect of IFN-*γ* therapy [5]. Awareness of the pronociceptive effect of IFN-*γ* from lumbar disc herniation is required [6]. Studies reveal that IFN-*γ* releases during the disc herniation process. It affects and regulates circulating macrophages in bone marrow and in disc tissue on a degenerated level [7]. Some endogenous cytokine production by glial cells and neurons has been detected [8]. IFN-*γ* induces hyperexcitability of spinal dorsal horn neurons in vitro, indicating a potential role of proinflammatory cytokines in central sensitization. IFN-*γ* induces long-lasting depolarization of inhibitory loops thereby sensitizing the nociceptive ascending pathways [9]. A significant increase in C-fiber response following application of disc material and administration of IFN-*γ* onto dorsal nerve roots was observed [6]. Rapid nociceptive effect after application of IFN-*γ* onto dorsal nerve roots indicates direct action on ion channels [10]. A certain dose of IFN-*γ* may induce strong allodynia effect on rats [11]. Prolonged increased intrathecal levels of IFN-*γ* lead to the disruption of the normal GABAergic tone in the rats' spinal dorsal horn which contributes to pain development [12]. Additionally, prolonged stimulation of IFN-*γ* possibly induces neuronal dysfunction by the receptor-GluR1 complex leading to neuropathic pain [13, 14]. Recent data has shown synergic interplay between IFN-*γ* and TNF-*α* promoting neuroinflammation [15]. IFN-*γ* released from macrophages in nucleus pulposus during herniation and extrusion could reach to dorsal horns and affects inhibitory interneurons crucial for nociception [13, 14, 16]. Moen et al. indicate that IFN-*γ* contributes to pathogenesis in acute lumbar radicular pain following disc degenerative processes [6]. Interaction of various cytokines including IFN-*γ* in the area of nerve roots lasting several months may play an important role in the development of persistent pain and disability in patients with lumbar discopathy.

**Figure 1:**
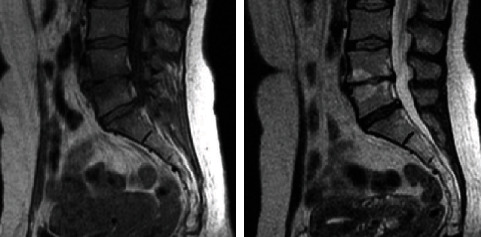
Modic type 1: decreased T1 signal and increased T2 signal of terminal lamina are visible at the L4-L5 level.

**Figure 2:**
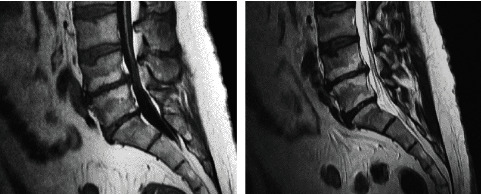
Modic type 2: increased T1 signal and increased T2 signal of terminal lamina are visible at L4-L5 and L5-S1 levels.

**Figure 3:**
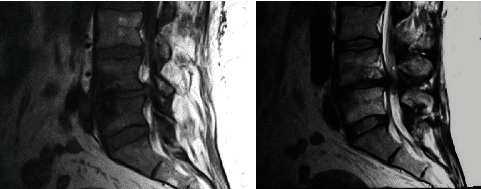
Modic type 3: decreased T1 signal and decreased T2 signal of terminal lamina are visible at the L4-L5 level.

**Figure 4:**
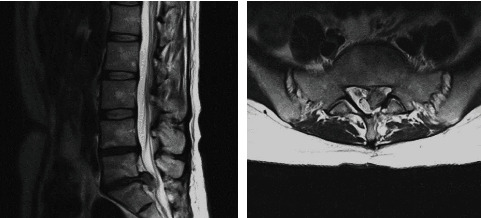
Intervertebral disc extrusion visible in the MRI.

Challenging IFN-*γ* serum levels with pain score scales combined with radiological scales such as Modic (Figures 1–3), clinical examination, and radiological findings (such as that visible in Figure 4) could bring new insight into the matter of postoperative pain relief. Microdiscectomy as a safe and common neurosurgical intervention resolving severe sciatica pain requires objective parameters to correlate with. The predictive value of the IFN-*γ* serum level is taken under consideration comparing pre- and postoperative results.

## 2. Materials and Methods

This prospective study was conducted among consecutive 27 patients, who met study inclusion criteria and were eligible for surgery because of sciatica pain due to lumbar disc herniation. The inclusion criteria were age between 18 and 70 years and the presence of symptomatic single-level lumbar disc herniation concerning the L4/L5 or L5/S1 level. The diagnosis was confirmed by magnetic resonance imaging (MRI). In MRI scans, the grade of disc degeneration according to the Pfirrmann classification, the severity of facet joint disease according to the Weisshaupt scale, and Modic changes were evaluated. Disc rupture and nerve compression were confirmed according to radiological findings and intraoperative view. Divergent patients were excluded. An uninterrupted conservative treatment period oscillated between 8 weeks and 12 months before surgery and proved to be ineffective. Exclusion criteria were the use of corticosteroid therapy preceding 3 months before surgery, previous spine surgery, spinal stenosis, rheumatoid diseases, diabetes, cancer, psychiatric diseases, recent surgery, pregnancy, alcohol or drug abuse, and cigarette smoking. All included patients underwent the procedure by standard microdiscectomy and were operated by an experienced surgeon. All patients were assessed (blood sampling, pain index forms) before operation and followed up with a check-up 3 months after the operation. All patients were subjected to standard physical and neurological examination. The straight leg raising test and sensory, motor, and reflex deficits were assessed. Additionally, results in the Numeric Rating Scale (NRS) for the back and legs, the Oswestry Disability Index (ODI), the Pain Rating Index (PRI) and Present Pain Intensity (PPI) of Short-Form McGill Pain Questionnaire (SF-MPQ), and Beck Depression Inventory (BDI) just 1 day before the operation were collected.

Indispensable to conducting the study, venous blood was collected in the morning and immediately centrifuged. Serum was stored at -80°C. A commercial enzyme-linked immunosorbent assay (ELISA) kit (Human IFN gamma Quantikine ELISA Kit, R&D) was used for plasma samples. We followed the protocol recommended by the manufacturer. The ELISA Reader Victor (PerkinElmer, USA) was used.

Statistical analyses were performed with the use of the IBM SPSS Statistics statistical package (version 24.0). The results are described as the mean, standard deviations, median values, and minimum and maximum values. Analysis of the differences between the level of interferon evaluated before and after the use of neurosurgical treatment was carried out with the use of the nonparametric Wilcoxon signed-rank test. For intergroup comparisons, we used the nonparametric Mann-Whitney *U* test for two independent samples. Rank correlation coefficients (*r*_*c*_ and *r*_*g*_) were used to assess the effect size. The *r*-Spearman rank correlation coefficient was applied to assess the relationship between variables. The significance level *α* = 0.05 was assumed in the analyses.

The study was approved by the Local Human Research Ethics Committee. All participating patients were included following the signature of the written informed consent statement.

## 3. Results

A group of 27 patients aged 20-56 participated in the study (*M* = 39.4; SD = 11.2), 10 women and 17 men. The vast majority of 18 people (66.7%) work in the physical profession, the rest in the mental profession. The average duration of pain lasted for *M* = 24.44 months (SD = 25.3) and ranged from 2 to 96 months (Table 1).

The analysis shows that the mean level of IFN-*γ* in patients after surgery increased (*Z* = −2.37; *p* = 0.017)—before the procedure, the mean value was 8.25 (SD = 5.18), after surgery, *M* = 9.61 (SD = 4.68); the effect size *r*_*c*_ = 0.46 indicates a moderate relationship between IFN-*γ* level and surgery (Table 2).

### 3.1. Clinical Assessment of the Pain and Disability

NRS results before and after surgery differ significantly: for NRS back: *Z* = −2.180; *p* = 0.029 and for NRS leg: *Z* = −3.968; *p* < 0.001. A significantly greater decrease in pain occurred in the area of the lower limb (*r*_*c*_ = 0.78) than in the back area (*r*_*c*_ = 0.44). The results in the ODI scale before and after the surgery differ significantly: *Z* = −3,600; *p* < 0.001; *r*_*c*_ = 0.7. The results in the pain indexes before and after the surgery differ significantly: for PRI: *Z* = −3.254; *p* = 0.001; *r*_*c*_ = 0.66 and for PPI: *Z* = −2.888; *p* = 0.004; *r*_*c*_ = 0.57. The reduction in scale results after the procedure indicates a significant weakening of pain (Table 2).

### 3.2. Dependencies between the IFN-*γ* Level and the Assessment of Pain

There are significant correlations between the results of the pain in the back region assessment NRS back scale after the surgery with the level of IFN-*γ* before the procedure (*r*_*s*_ = 0.528; *p* = 0.008) and after the procedure (*r*_*s*_ = 0.455; *p* = 0.025). The decrease in pain scores after surgery is correlated with the relatively lower IFN-*γ* levels postoperatively. There are significant correlations between the results of the PRI scale and the IFN-*γ* level. The PRI score before surgery correlates positively with IFN-*γ* after surgery (*r*_*s*_ = 0.462; *p* = 0.023), and the PRI score after surgery correlates positively with IFN-*γ* before surgery (*r*_*s*_ = 0.529; *p* = 0.005) and after surgery (*r*_*s*_ = 0.549; *p* = 0.003). All correlations are moderate in severity. There are no significant relationships between the results of the ODI scale and the IFN-*γ* level. On the verge of significance is the low positive correlation between ODI scores before surgery and the IFN-*γ* level before surgery (*r*_*s*_ = 0.376; *p* = 0.058)—high ODI results are accompanied by an increase in the IFN-*γ* level. There are no significant correlations between the Beck Depression Scale and the IFN-*γ* level (Table 3).

### 3.3. Analysis due to the Presence of Neurological Symptoms

In the clinical group, 16 patients (59.3%) outlined a painful straight leg raising test, 18 patients (66.7%) reported reflex disorders, and 8 (29.6%) had paresis. In further analyses, it was checked whether the level of IFN-*γ* varies depending on the occurrence of neurological symptoms. There were no statistically significant differences in the level of IFN-*γ* in patients with neurological symptoms (straight leg raising test, reflex disorder, and paresis) and without these symptoms (Table 4).

### 3.4. Analysis due to Radiological Signs and Level of IFN-*γ*

The comparison of the IFN-*γ* level in the groups of patients with and without the problem of ruptured disc showed no statistically significant differences before surgery (*Z* = −0.794; *p* = 0.427), as well as after surgery (*Z* = −1.323; *p* = 0.186). Noteworthily, there is not only a higher average but also a much greater differentiation of the IFN-*γ* level in the group of patients with a ruptured disc than in the group with an unruptured disc. There were significant differences in the IFN-*γ* level before (*Z* = −2.733; *p* = 0.006; *r*_*g*_ = 0.89) and after (*Z* = −2.391; *p* = 0.017; *r*_*g*_ = 0.78) surgery in the groups of patients with and without nerve compression. In the group of patients with nerve compression, the level of IFN-*γ* before and after surgery was lower. There is a strong relationship between the nerve compression and the IFN-*γ* level. We identified 14 cases of Modic changes of type 2. The IFN-*γ* level before the procedure in patients with positive Modic changes differs significantly from patients with negative Modic changes—*Z* = −2.465; *p* = 0.014; *r*_*g*_ = 0.64 (Table 5).

According to the Pfirrmann scale, there were 4 patients with grade two, 13 with grade three, and 10 with grade four of disc degeneration. According to the Weisshaupt scale, there were 12 patients with normal facet joint space (grade 0), 2 with grade one, 8 with grade two, and 5 with grade three. The analyses did not show a statistically significant relationship between the IFN-*γ* level and the results of the scale of disc degeneration and the degree of osteoarthritis (Table 6).

## 4. Discussion

The study was carried out to investigate the role of the IFN-*γ* serum level in patients with lumbar radiculopathy treated with lumbar microdiscectomy, to examine correlations of IFN-*γ* with neurological symptoms, and to inspect relation with radiological signs and scales, before and after the procedure. A rare similar research has been performed on humans in this area. There are some limitations. The studies measuring the concentration of IFN-*γ* in the area of the nerve root or in the cerebrospinal fluid (CSF) were not performed yet so there is no evidence regarding the influence of IFN-*γ* on nerve roots or spinal cord pathways. There were ineffective efforts to evaluate cytokine concentrations in epidural space [17]. Local application of IFN-*γ* on nerve roots in animals did not cause reduction in nerve conduction velocity [18]. Better results were achieved when measuring the IFN-*γ* level in the cerebrospinal fluid (CSF) after pulsed radiofrequency treatment in patients with radicular pain [4]. The necessity of lumbar puncture is considered a serious disadvantage such as pain monitoring, so searching for neuroimmune mediators in serum is believed to be safer and more useful.

Determining the role of interferons in pain processes seems to be essential. Knowledge about pain processes concerning the molecular level seems to be a promising target for analgesic therapy, especially in the treatment of neuropathic pain. The animal model of a chronic constriction injury (CCI) of nerve structures causing neuropathic pain used to be used by many researchers [19–22]. Sciatica patients with compression of nerve roots in the course of lumbar disc herniations are often considered as the representative of neuropathic pain due to neuroinflammation and nerve damage [23]. To sum up, sciatica pain is not an example of pure neuropathic pain; there is a prominent component of neuroinflammation and nociceptive pain associated with mechanical nerve compression. Nevertheless, the group of discopathic patients was chosen to evaluate interferon interaction because of its potential to homogeneity and possibility in clear detection of the pain source.

The core of the above analysis seems to be the evaluation of the IFN-*γ* serum level changes in patients before and after surgery. Without additional causes of pain and comorbidities targeting IFN levels, the period of 3 months after surgery was chosen for the assessment to avoid abbreviations connected with healing of the postoperative wound. This is the time in which the patient can return to normal life and work after initial rehabilitation.

The analysis shows that the level of IFN-*γ* in patients after surgery increased significantly compared to the preoperative level with simultaneous reduction of pain. This is due to the fact that the surgery first removes the compression on the nerve root without affecting the ongoing process of neuroinflammation. Neuroinflammation decreases gradually after regression of the nerve root compression; some of the painful symptoms may persist after the surgery despite the end of the mechanical injury of the root. There is a question of whether the incision of the disc annulus and the exposure of the disc material can be a source of IFN-*γ* leading to its increase in serum [7, 24, 25]. Interestingly, low pain scores after surgery (in the assessment by NRS back and PRI) correlate positively with the IFN-*γ* level before surgery and after surgery—reduction of pain after surgery correlates with the serum level of IFN-*γ*.

The analysis points at no significant differences in the IFN-*γ* level were found in individuals with an increased straight leg rising test symptom (≤45°) and less severe (>45°). It may be related to the fact that the straight leg raising test is a clinical expression of mechanical nerve compression, and, as a result, it is mostly an indicator of nociceptive pain [26].

The level of interferon in the group of patients with a ruptured disc and without shows no significant differences both before and after the surgery. However, attention is drawn to the fact that in the group with the ruptured disc, there is a higher average of serum interferon level as well as its much greater variation. It may be related to the length of exposure of the disc material of the nucleus pulposus. It is worth remembering that an unruptured disc's nucleus pulposus is isolated from the circulatory system [24, 27, 28].

According to Pfirrmann, we can distinguish a few variants of nerve root compromise by disc herniation. There are no contact, deviation, and nerve compression [29]. There were significant differences in the level of IFN before and after surgery between the groups of patients with compression and without nerve compression in the MRI. In the group of patients with the lack of nerve compression, the level of IFN before and after surgery was higher. The higher serum IFN-*γ* level, despite the lack of significant nerve compression, may be the expression of central sensitization leading to hyperalgesia [5].

Modic changes are vertebral endplate and surrounding bone marrow changes in MRI [30, 31]. They are considered as probably a disc degeneration-related process, and there are a few concepts about the background of these changes [32–34]. Han et al. described that high expression of IFN-*γ* was detected in the group with the embedment of nucleus pulposus into subchondral bone in an animal model [35]. In our study, the serum level of IFN-*γ* in patients with positive Modic changes is significantly smaller than that in patients without confirmed Modic changes. The role of IFN-*γ* and Modic changes is still unclear.

Elevated levels of many interleukins accompanied spondylolisthesis and disc herniation. Sutovsky et al. point at IFN gamma levels which were significantly higher in patients with spondylolisthesis and disc herniation in assessed intervertebral disc tissue compared to the control group. Levels of interferon gamma were significantly higher in the annulus fibrosus compartment comparing to nucleus pulposus levels (79.46 + −45.01 vs. 73.98 + −14.38). Results suggest the predominant expression of IFN gamma in the peripheral part of the degenerated disc. Close proximity between herniated annulus fibrosus and nerve roots could explain regional sensitization [36].

A study concerning neuropathic pain (NP) in the course of Failed Back Surgery Syndrome (FBSS) treated successfully with spinal cord stimulation (SCS) brings interesting results. Successful SCS did not influence elevated serum levels of IFN-*γ* which remains unchanged despite meaningful pain relief in patients suffering NP. Those findings suggest complex mechanisms underlying pain reduction during cord stimulation. Undoubtedly IFN-*γ* seems to be a key to understand pain processes [37].

## 5. Conclusions

Clinical assessment of pain (NRS scale, PPI scale) after operation positively correlates with a lower level of IFN-*γ*. In the group of patients without significant nerve compression confirmed by MRI investigation, the level of IFN-*γ* before and after surgery was higher than that in the group with nerve root compression. Because IFN-*γ* may be a target of therapy, further investigations are needed to establish the role of IFN-*γ* in raising and maintenance of sciatic pain.

## Figures and Tables

**Table 1:** Characteristics of patients.

Number of patients	Age		Gender				Duration of pain (months)		Extent of herniation				Disc herniation level				IFN-*γ* before surgery (pg/ml)	IFN-*γ* after surgery (pg/ml)
	Mean ± SD	Range	Male		Female		Mean ± SD	Range	Protrusion		Extrusion		L4-L5		L5-S1		Mean ± SD	Mean ± SD
			(*n*)	(%)	(*n*)	(%)			(*n*)	(%)	(*n*)	(%)	(*n*)	(%)	(*n*)	(%)		
27	39 ± 11	25-56	17	63	10	37	24 ± 25	2-96	10	37	17	63	10	27	17	63	8.25 ± 5.18	9.61 ± 4.68

**Table 2:** Subjective pain/disability evaluation and IFN-*γ* level before and after surgery.

		Before surgery	After surgery	*p*
		Mean ± SD	Mean ± SD	
NRS back	**0-10**	3.58 ± 3.91	2.08 ± 2.73	**0.029**
NRS leg	0-10	5.00 ± 2.99	0.85 ± 1.78	**<0.001**
PRI	0-35	14.75 ± 11.90	8.67 ± 10.19	**0.001**
PPI	0-5	2.23 ± 1.21	1.33 ± 1.00	**0.004**
ODI	0-50	23.77 ± 8.47	14.78 ± 9.54	**<0.001**
IFN-*γ*	pg/ml	8.25 ± 5.18	9.61 ± 4.68	**0.017**

NRS back and leg: Numeric Rating Scale; PRI: Pain Rating Index; PPI: Present Pain Intensity; ODI: Oswestry Disability Index; Wilcoxon rank test for dependent samples with *p* < 0.05 highlighted in bold.

**Table 3:** Correlation between the IFN-*γ* level and the subjective assessment of pain.

		IFN-*γ* before surgery (pg/ml)	IFN-*γ* after surgery (pg/ml)
		*r*_*s*_ (*p*)	*r*_*s*_ (*p*)
NRS back	Before	0.247 (0.244)	0.34 (0.104)
	After	0.528^∗∗^ (0.008)	0.455^∗^ (0.025)
NRS leg	Before	-0.029 (0.886)	0.065 (0.748)
	After	0.233 (0.251)	0.090 (0.663)
PRI	Before	0.401^g^ (0.052)	0.462^∗^ (0.023)
	After	0.529^∗∗^ (0.005)	0.549^∗∗^ (0.003)
PPI	Before	0.273 (0.176)	0.277 (0.171)
	After	0.283 (0.152)	0.263 (0.186)
ODI	Before	0.376^g^ (0.058)	0.227 (0.266)
	After	0.145 (0.472)	0.107 (0.595)

^∗^Correlation significant at *p* < 0.05; ^∗∗^correlation significant at *p* < 0, 01. NRS back and leg: Numeric Rating Scale; PRI: Pain Rating Index; PII: present pain intensity; ODI: Oswestry Disability Index; Wilcoxon rank test for dependent samples with *p* < 0.05 highlighted in bold.

**Table 4:** Neurological findings and IFN-*γ* level.

Neurological findings		*n*	IFN-*γ* before surgery (pg/ml)	*p*	IFN-*γ* after surgery (pg/ml)	*p*	
			Mean ± SD		Mean ± SD		
Straight leg raising test	0-45°	16	8.48 ± 6.67	0.236	9.68 ± 6.10	0.805
	>45°	11	7.90 ± 1.66		9.51 ± 1.09	
Hyporeflexia	Yes	18	8.06 ± 6.26	0.071	8.80 ± 5.30	0.057
	No	9	8.62 ± 1.87		11.23 ± 2.69	
Reduced muscle strength	Yes	8	10.82 ± 8.61	0.750	11.22 ± 6.27	0.750
	No	19	7.16 ± 2.39		8.93 ± 3.84	

**Table 5:** Radiological findings (MRI) and IFN-*γ* level.

Radiological findings			IFN-*γ* before surgery (pg/ml)		IFN-*γ* after surgery (pg/ml)	
		*n* ^∗^	Mean ± SD	*p*	Mean ± SD	*p*
Disc	Unruptured	10	6.36 ± 1.67	0.427	7.99 ± 2.30	0.186
	Ruptured	17	9.54 ± 7.41		10.70 ± 11.88	
Modic changes	Yes	19	5.91 ± 1.63	**0.014**	7.85 ± 3.84	0.055
	No	8	11.92 ± 8.16		12.31 ± 6.03	
Nerve compression	Yes	23	6.18 ± 2.15	**0.006**	8.22 ± 4.30	**0.017**
	No	4	16.72 ± 8.98		15.08 ± 5.15	

*n* ^∗^: 5 patients had MRI and intraoperative divergences so were missed out. Mann-Whitney *U* test for two independent samples with *p* < 0.05 highlighted in bold.

**Table 6:** Radiological assessment of disc and facet joint degeneration.

Scales	Grade	*n*	%
Pfirrmann grading	Grade 2	4	15
	Grade 3	13	48
	Grade 4	10	37
Weisshaupt grading	Grade 0	12	44
	Grade 1	2	7
	Grade 2	8	30
	Grade 3	5	19

## Data Availability

The data used to support the findings of this study are available from the corresponding author upon request.
